# Exogenous attention to social stimuli in the neurotypical population: The impact of autism traits

**DOI:** 10.1192/j.eurpsy.2021.1632

**Published:** 2021-08-13

**Authors:** F. Barros, N. Teixeira, C. Figueiredo, S. Silva, S. Soares

**Affiliations:** 1 William James Center For Research (wjcr), Department Of Education And Psychology, University of Aveiro, Aveiro, Portugal; 2 Center For Health Technology And Services Research (cintesis), Department Of Education And Psychology, University of Aveiro, Aveiro, Portugal; 3 Research Unit On Governance, Competitiveness And Public Policies (govcopp), University of Aveiro, Aveiro, Portugal; 4 Institute Of Electronics And Informatics Engineering Of Aveiro (ieeta), University of Aveiro, Aveiro, Portugal; 5 Department Of Electronics, Telecommunications, And Informatics (deti), University of Aveiro, Aveiro, Portugal

**Keywords:** autism, Attention, social cognition, Broader Autism Phenotype

## Abstract

**Introduction:**

Autism Spectrum Disorders (ASD) have been associated with decreased spontaneous attention to social stimuli. Several studies further suggest that a higher expression of autism traits (AT) in the neurotypical population (NTP) may also be related to decreased social attention, although the evidence is still scarce, especially when considering faces as task-irrelevant distractors.

**Objectives:**

This study aimed to explore the relationship between the expression of AT in the NTP and exogenous attention to social stimuli.

**Methods:**

Fifty-one adult participants were recruited and asked to complete the Autism Spectrum Quotient (AQ), to measure AT, and to perform an attentional capture task. In the latter, they were instructed to detect a target letter in the middle of perceptually similar (high perceptual load) or dissimilar (low perceptual load) distractor letters. In 25% of the trials, task-irrelevant distractors, consisting of images of faces (social) or houses (non-social), were shown flanking the letter stimuli.

**Results:**

Response times were found to be affected by distractor-response compatibility, increasing for contralateral distractors, but decreasing for ipsilateral distractors, in relation to trials with no distractors (baseline). Importantly, these trends were magnified for distractor faces in the group with less AT, considering the social skills dimension of AQ, while the same tendency was observed in the group with higher AT, but for distractor houses.

**Conclusions:**

Our results support an altered attentional performance in the subclinical phenotype of the autism spectrum. Furthermore, they also add to existing literature documenting similar attentional abnormalities in both the clinical and subclinical extremes of the spectrum, hinting possible shared mechanisms.

**Disclosure:**

No significant relationships.

